# Diurnal rhythm of urinary aquaporin-2 in children with primary monosymptomatic nocturnal enuresis

**DOI:** 10.55730/1300-0144.5780

**Published:** 2023-11-27

**Authors:** Nehal ABDELHAMID, Mohamed A ABD ALMAWLA, Aliaa A WAHBY, Mervat ISMAIL, Hani Abdelsalam ELMIKATY, Hanan M HAMED, INGY ASHMAWY, Batoul Mohamed ABDELRAOAF, Enas Mokhtar ABDELHAMID, Hassan M SALAM

**Affiliations:** 1 Departments of Pediatrics, Medical Research and Clinical Studies Institute, National Research Centre, Cairo, Egypt; 2 Department of Clinical Pathology, Medical Research and Clinical Studies Institute, National Research Centre, Cairo, Egypt; 3 Departments of Pediatrics, Ainshams University, Cairo, Egypt

**Keywords:** Aquaporin 2, vasopressin, nocturnal enuresis

## Abstract

**Background/aim:**

Nocturnal enuresis can be frustrating for children and their families as the child ages. Our aim is to evaluate urine aquaporin 2 (AQP-2) as a noninvasive biomarker of water balance in children with primary monosymptomatic nocturnal enuresis (PMNE).

**Material and methods:**

The study included 90 children; sixty-eight children suffering from PMNE aged (9.57 ± 2.16) years and 22 healthy children with good toilet control, matched sex and age. All enuretic children were subjected to complete history taking, clinical evaluation, and bed wetting diary. Serum arginine vasopressin (AVP) and urine AQP-2 were tested in the morning (at 9–11 am) and evening (at 9–11 pm). Blood urea, creatinine, Na, glucose, urine osmolality, Ca/Cr, Alb/Cr and specific gravity were tested simultaneously.

**Results:**

Serum AVP, urine AQP-2, and urine osmolality were statistically lower in patients than controls. Patients had a significantly lower level of night serum AVP concentrations, urine AQP-2, and urine osmolality than the corresponding morning level. Urine AQP-2 was significantly correlated with urine osmolality (p < 0.05). AQP-2 had a sensitivity of 90% and a specificity of 70%. However, no statistically significant correlation was found between serum AVP and urine AQP-2.

**Conclusion:**

Primary monosymptomatic nocturnal enuresis in children could be associated with reduction of urine excretion of AQP-2 at night. Urine AQP-2 is significantly correlated with urine osmolality. Therefore, it may be a noninvasive biomarker of hydration status in children with PMNE, with good sensitivity and specificity.

## 1. Introduction

Primary monosymtomatic nocturnal enuresis (PMNE) is a frequent developmental problem of childhood, which often has a similar condition within the family, and leads to psychological and social problems for the child and his family [1]. It affects about 15%–20% of toddlers aged 5, 10% of 7–12 year olds and up to 2% of grown up [2].

Enuresis is named primary when the child has not had dry nights for six months [3]. Most cases of NE (85%) are of the primary type [4]. There are two types of enuresis: monosymptomatic (MNE) neither associated with lower urinary tract symptoms nor with bladder dysfunction (approx. 80% of children with NE); and nonmonosymptomatic (NMNE), related to symptoms suggesting lower urinary tract infection such as urgency, frequency, diurnal incontinence, and/or dysuria (approx. 20% of children with NE)[5]. Different factors may influence PMNE including deep sleep, a shift in family dynamics, a family history of NE, small functional capacity of the bladder, and overactive bladder. Immaturity and genetic impact may play a part in the persistence of PMNE [6].

The antidiuretic hormone (ADH) which is secreted from posterior pituitary in normal children, increases at night as a result of bladder distension in attempt to reduce urine production [7]. This normal circadian rhythm in ADH secretion, i.e. arginine vasopressin (AVP) may be absent in patients with PMNE [8]. Loss of circadian rhythm of AVP leads to lack of the adequate influence by the molecular clock. These children generate immense amounts of diluted urine during the night of which exceeds urinary bladder volume, leading to bed wetting [9]. In PMNE, urine overproduction at night, associated with inadequate secretion of AVP, detrusor muscle overactivity and sleep disorders play key roles. Regardless of pathophysiology, the main therapeutic modalities of PMNE are alarm clock treatment and desmopressin(DDAVP), while the tricyclic antidepressants are used for refractory cases. One main reason is that clarifying the precise cause of enuresis is hard [10] because accurate evaluation of urinary bladder volume and polyuria at night by utilizing a frequency volume chart is hard with little ones that struggle with bedwetting. Parents are obliged to purchase suitable diapers and an accurate digital scale in order to weigh the diapers and measure nocturnal urine output. Vasopressin is the main hormone involved in water homeostasis, essential for life. It targets the V2 receptor, within the renal collecting duct, promoting H2O reabsorption through activation of aquaporin-2(AQP-2) [11]. Measurements of circulating vasopressin was found to be difficult and with a wide laboratory error, as most of it (More than 90%) binds to platelets and decomposes rapidly in blood samples. AQP2 is involved in the urinary concentration in collecting ducts. It mediates transmembrane water transport in the collecting ducts [12,13]. It is consistently important to evaluate urine AQP-2 rhythm, as a useful noninvasive biomarker of vasopressin function in children with PMNE.

## 2. Material and methods

### 2.1. Subjects

The study protocol was approved by the institute ethics board of the National Research Centre, Cairo, Egypt [committee approval code no:12060133#1]. The study conforms to the provisions of the Declaration of Helsinki (2008). Informed consents were taken from the parents and/or caregivers before carrying out the procedure.

The present study involves 90 children. Sixty-eight children (40 males and 28 females) aged 9.57 ± 2.16 suffering from primary monosymptomatic nocturnal enuresis (at least three wet nights weekly), and 22 healthy children, with good toilet control day and night, matched for sex and age were selected as the control group. The selection of cases follows the updated standardization document of the International Children’s Continence Society [14] that primary monosymptomatic nocturnal enuresis takes place within kids that were not able to maintain dryness for six months excluding children with any lower urinary tract manifestations or a history of urinary bladder dysfunction. They were recruited randomly (between January 2020 and April 2021) by the pediatric outpatient clinic at the Medical Research and Clinical Studies Institute along with the National Research Centre in Cairo, Egypt. The blood creatinine, urine analysis, and urine culture were normal. Renal ultrasounds were without any residual urine (≤20 mL residual urine) in the postvoiding ultrasound and consequently absenteeism of neurological and urological disorders. Lumbosacral x ray image was done for spina bifida on all cases. None of them had received any desmopressin treatment for the past 6 months. The children were allowed to drink 2 h before going to sleep and were asked to void before sleeping.

All enuretic children were subjected to a bedwetting diary should be completed, documenting wet nights, for 7 consecutive nights. History to exclude any coexisting condition such as diabetes mellitus or insipidus, encopresis, and chronic constipation. Lower Urinary tract symptoms were assessed (urgency, frequency, dribbling, burning, daytime incontinence). History of parental enuresis during childhood and consanguinity, difficulty arousal from sleep, history of major stressful events such as a shift in family dynamics (e.g., the birth of a sibling, a divorce), and clinical evaluation of adenotonsilar hypertrophy were all recorded.

### 2.2. Methods

The Laboratory investigations in the children were measured in serum and urine samples were collected during the morning (9–11 am) and evening (9–11 pm) as follows:

1. Blood glucose, urea nitrogen, and sodium levels were documented to calculate the blood osmolality values of the patients. The Worthley equation formula (Osmolality = 2*[Na+] + BUN (mg/dL)/2.8 + Glucose (mg/dL)/18) was accepted as blood osmolality calculations were made with this formula in the present study [15].
2. Serum assessment of vasopressin was evaluated, following centrifugation, sera were stored at −20 Celsius until evaluation. Vasopressin was assayed in the obtained serum samples using human neurophysin II/Arg-vasopressin an enzyme immunoassay kit (colorimetric) from Novus Biologicals, Centennial, USA. The kit utilizes polyclonal antibody to vasopressin to bind in a competitive manner. Detection range 15.63–1000 pg/mL and sensetivity of 9.38 pg/mL.
3. Urine samples were tested for urine osmolality (mOsm/kg H2O) using an automatic osmometer and urine aquaporin-2 (AQP2) concentration was assayed by a direct ELISA employing rabbit-anti-AQP2 antibody (Novus Biologicals, Centennial, USA) with a lower level of identification of 0.94 ng/mL and intra-assay coefficient of variation of 6.1%.

### 2.3. Statistical analysis

Statistical package for the social sciences (SPSS, version 18.0 for Windows; SPSS Inc., Chicago Illinois, USA) was utilized for statistics. Quantitative variables were demonstrated as means ± standard deviation (SD). Variables were expressed as frequencies and percentages. An Independent t-test was applied in order to compare the means of variables for 2 unrelated groups. A paired sample t-test was used to compare means of 2 variables within a group (morning and night) Pearson correlation was used to calculate the pairwise associations for a set of variables. The p values ≤ 0.05 were considered significant. Receiver operating characteristic (ROC) curve was generated and the area under the curve (AUC) was evaluated. An AUC of 0.5 is no better than expected by chance, whereas a value of 1.0 signifies a perfect biomarker [16].

## 3. Results

Demographic data of the cases and controls are illustrated in Table 1. The mean values for pre and postvoiding urine were 202.696 ± 57.41 and 8.60 ± 11.24 mL respectively.

### 3.1. AVP

Patients had a significantly lower level of night serum AVP concentrations than corresponding morning levels (p < 0.05) as shown in Table 2 while, vasopressin rhythm was reversed day/night (D/N) rhythm in 57/68 (84%) of cases. Meanwhile, the morning level of AVP was statistically higher in patients than control group (691.9 ± 572.6 versus 70.9 ± 34.3 pg/mL) (p < 0.001).

### 3.2. Osmolality

In patients, Morning urine osmolality was lower than corresponding night results with a statistically significant difference (p < 0.05) (Table 2).Meanwhile, the D/N rhythm was reversed at 57%. However, urine osmolality was found statistically significant when compared to control at morning urine samples only (Table 3). No significant difference was found between morning and night serum osmolality levels in patients (Table 2).

### 3.3. Urine AQP- 2

Patients had a significantly lower level of night urine AQP-2 than corresponding morning levels (p < 0.001) (Table 2). Meanwhile, AQP-2 D/N rhythm was reversed in 54/68 (79%) of patients. However, no significant correlation was found between serum AVP and corresponding AQP-2 in patients (Tables 4a, 4b).

Meanwhile, Morning urine AQP-2 was significantly correlated with Ca/Cr and Alb/cr ratios, serum Na, and serum osmolality. While Night values correlated with serum glucose at p < 0.05. Significant positive correlations were found between morning and night urine AQP-2 and corresponding urine osmolality (Tables 4a, 4b-Figures 1a,1b).

Compared to healthy children, urine AQP-2 were statistically lower in patients than controls at both morning and night values (p < 0.001) (Table 3).

**Figure 2:**
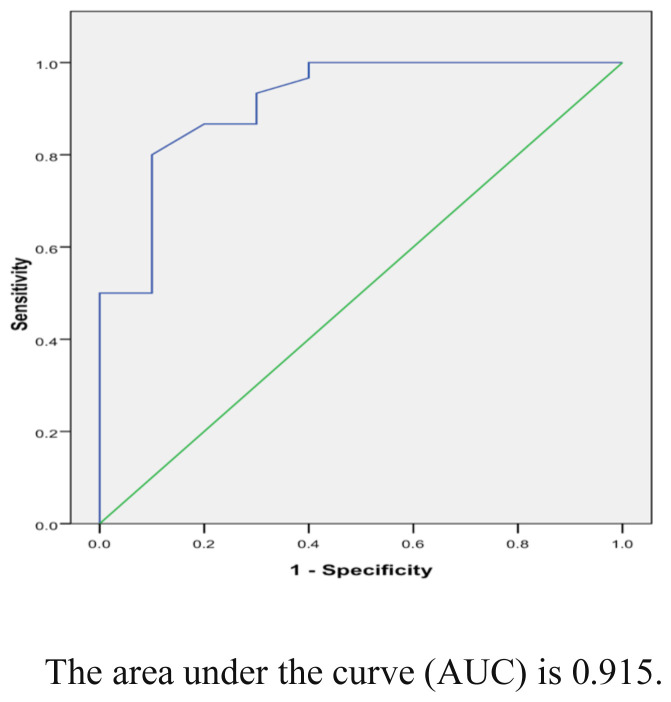
Receiver operating-characteristic (ROC) of urine AQP-2 in patients with PMNE.

ROC curve of the AQP-2 is shown in Figure 2 with the area under curve (AUC) of 0.915, sensitivity of 90%, and specificity of 70%.

## 4. Discussion

The principal role of the pituitary arginine vasopressin is to adjust water reabsorption by V2 receptor-mediated recruitment of aquaporins in the collecting ducts of the kidney, and hence maintaining blood osmolality within a narrow range [17]. Arginin vasopressin binds to the V2 receptor expressed in the basolateral side of the principal cells in the renal collecting duct. This activates the adenylate cyclase and produces cyclic adenosine monophosphate. The latter triggers protein kinase that phosphorylates the *C* terminal residues of AQP-2. Thus AQP-2 is positioned into the apical side of the principal cell forming AQP-2 water channels. H2O is reabsorbed by these channels into the principal cells and exits through AQP-3 and AQP-4 water channels to the interstitium. Thus, H2O is reabsorbed into the blood [18,19].

The serum vasopressin level in normal subjects has a characteristic circadian rhythm, which increases at night. However, for children with PMNE, this rhythm could be lost, with no significant peak level at night [20].

After the release of AVP, only 10–20% of the sum remains in the blood stream because its plasma half-life is only 6–20 min [21]. Therefore, we measured the variability of the day/night rhythm of AVP and urine AQP-2, as a marker reflecting AVP profiles. Patients had a significantly lower level of night serum AVP and urine AQP-2 concentrations than corresponding morning levels. Meanwhile, AVP and AQP-2 D/N rhythms were reversed in 84% and 79% respectively. Compared to normal subjects’ urine AQP-2 was statistically lower.

Similar to our results Radetti et al. [22] showed that night level of urine AQP-2 was lower in nocturnal enuresis patients, but statistical significance was only found in DDAVP responders. Valenti et al., [23] calculated D/N ratios of urine AQP-2 in their cases and found that these ratios were increased in cases of NE. In addition, Hara et al. [24] found that after eight weeks of vasopressin intake, there was a significant correlation between the D/N ratio of AQP-2 and the percentage of wet nights. Meanwhile, there was a significant change in AQP-2 D/N ratio from before treatments to complete remission in responders.

In enuretic children, the reversed circadian rhythm of vasopressin levels directs the AQP-2 moving towards the apical membrane of collecting-duct principal cells. This explains that AVP regulates the AQP-2 expression in the collecting duct as well as that children with low serum AVP levels are more prone to having lowered expression of AQP-2.

In the present study, urine AQP-2 was statistically lower in cases than controls. Patients expressed significantly lower levels of night serum AVP concentrations, urine AQP-2, and urine osmolality than corresponding morning levels. Meanwhile, urine AQP-2 was significantly correlated with urine osmolality (p < 0.05) in both night and morning samples. Pomeranz et al. [25] revealed a decrease in urine osmolality proportional to a reduction in serum AVP in cases with NE.

This goes hand in hand with the observation that vasopressin levels regulate AQP2 excretion in the urine and hence, explains the significant positive correlations found between morning and night urine AQP-2 and corresponding urine osmolality. Based on this finding, we can interpret the tendency of the children suffering from nocturnal enuresis to exhibit lower concentrations of urine AQP-2 in comparison to healthy subjects.

Our findings seem to suggest that enuretic children tend to release lower amounts of AQP-2 as a result of low AVP stimulation, likely due to an immature or altered function at the receptor or postreceptor level. DDAVP treatment for nocturnal enuresis might increase AQP-2 production, leading to limited bed wetting. Meanwhile, a significant correlation between morning urine AQP-2 and corresponding serum sodium and serum osmolality was noticed. Sodium absorption is essential for the urinary concentrating capacity [24]. In fact, in healthy subjects, urine concentration results from the joined actions of the loop of Henle and the collecting duct. The loop of Henle generates high osmolarity within the renal medulla by the countercurrent multiplication system as the collecting duct, under the influence of AVP permits, osmotic equilibration between the urine and hypertonic interstitium. The generation of high osmolality in the medulla depends mostly on NaCl absorption due to the activity of the Na+ transporters situated in the thick ascending limb. AVP manages serum osmolality via V2 receptors and hence AQP-2 water channels of the renal epithelial cells of collecting ducts are stimulated with the V2- vasopressin receptor and therefore leads to water retention and hyponatremia. The V2-vasopressin receptor is expressed on collecting duct cells and on cells of the thick ascending loop of Henle of the nephron. In the latter (as well as in a section of collecting duct cells) the triggered V2 receptor enhances the reabsorption of sodium [26]. On the other hand, water intake for a normal individual suppresses AVP levels and reduces antidiuresis in response to decreased AQP-2 recruitment at the apical membrane of collecting duct cells. In consequence, less AQP2 is released into the urine [27,28].

## Conclusions

Therefore, we can conclude that nocturnal diuresis in children suffering from primary monosymptomatic nocturnal enuresis could potentially be a result of reversed circadian rhythm of vasopressin levels, which consequently regulates the AQP2 levels at renal collecting-duct cells. The reversed rhythm of urine AQP-2 can reflect inappropriate water balance in children with primary monosymptomatic nocturnal enuresis with good sensitivity and specificity. Further studies are necessary to investigate the value of urinary AQP2 as a marker to guide their treatment with DDAVP.

## Figures and Tables

**Figure 1a:**
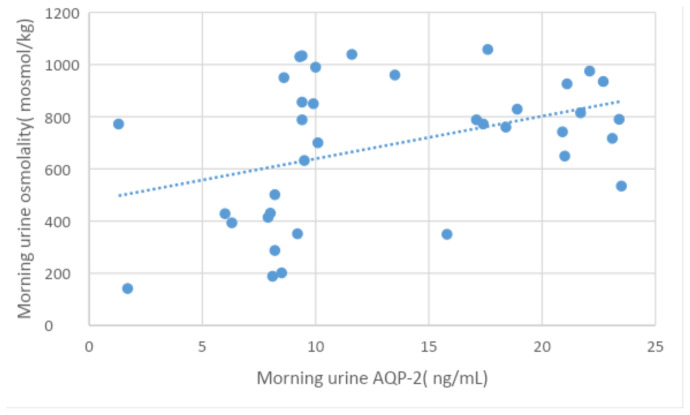
Correlation between morning urine AQP2 and morning urine osmolality in patients.

**Figure 1b:**
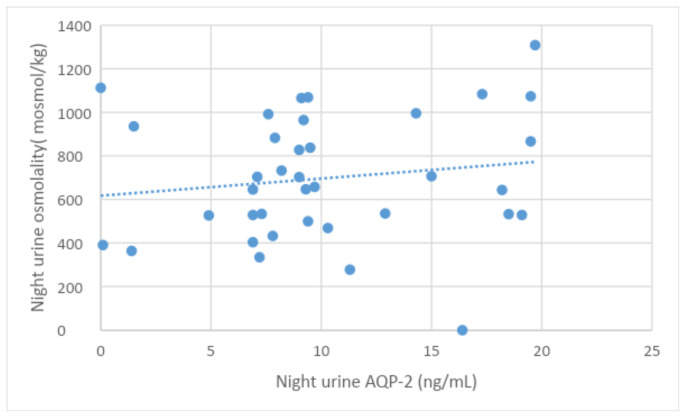
Correlation between night urine AQP2 and night urine osmolality in patients.

**Table 1:** Demographic data of studied children.

		Patients (n = 68)		Controls (n = 22)		p value
Variables		Frequency	Mean ± SD	Frequency	Mean ± SD	
Age (years)			9.57 ± 2.16		9.75 ± 2.1	0.7
Weight(Kg)			36.77 ± 14.5		37.71 ± 15.8	0.8
Height(m)			1.4 ± 0.14		1.39 ± 0.13	0.7
BMI			18.7 ± 4.08		18.4 ± 4.46	0.9
Sex	Male	40(58.8%)		13(59%)		
	Female	28(41.2%)		9(40%)		
Constipation	No	64(94.1%				
	Yes	4(5.9%)				
Pin worm	No	58(85.3%)				
	Yes	10(14.7%)				
Adenoids	No	48(70.6%)				
	Yes	20(29.4%)				
Difficult arousal from sleep	No	20(29.4%)				
	Yes	48(70.6%)				
Consanguinity	No	50(73.5%)				
	Yes	18(26.5%)				
Similar condition	No	16(23.5%)				
	Yes	52(76.5%)				
Spina bifida occulta	No	55(81%)				
	Yes	13(19%)				

**Table 2:** Comparison between day/night level of serum AVP, urine AQP-2,urine osmolality and serum osmolality in studied patients.

Variables	Mean ± SD	p value
Morning serum AVP (pg/mL)	744.094 ± 145.3	0.008*
Night serum AVP (pg/mL)	401.7 ± 63.1	
Morning urine AQP-2 (ng/mL)	13.3 ± 5.6	0.001**
Night urine AQP-2 (ng/mL)	9.8 ± 4.9	
Morning serum osmolality (mosmol/kg)	291.00 ± 5.61	0.2
Night serum osmolality (mosmol/kg)	296.16 ± 8.45	
Morning urine osmolality (mosmol/kg)	753.07 ± 27.87	0.03*
Night urine osmolality (mosmol/kg)	674.17 ± 32.77	

* P value is significant at the 0.05 level

** P value is highly significant at the 0.001 level

**Table 3:** Comparison between day and night level of urine AQP-2 and urine osmolality between patients and control groups.

Variables		Mean ± SD	p value
Morning urine AQP-2 (ng/mL)	Patient	13.3 ± 5.6	0.001**
	Control	19.9 ± 1.2	
Night urine AQP-2 (ng/mL)	Patient	9.8 ± 4.9	0.001**
	Control	17.1 ± 1.9	
Morning urine osmolality(mosmol/kg)	Patient	753.07 ± 27.87	0.04*
	Control	541.36 ± 87.422	
Night urine osmolality( mosmol/kg)	Patient	674.17 ± 32.77	0.3
	Control	696.5 ± 236.8	

* P value is significant at the 0.05 level

** P value is highly significant at the 0.001 level

**Table 4a:** Correlation between morning serum AVP, urine AQP-2 and studied parameters in patients.

Variables		Morning serumAVP	Morning urine AQP-2
Morning serum AVP	Pearson correlation	1	−.016
	Sig. (2-tailed)		.923
Morning urine AQP-2	Pearson correlation	−.016	1
	Sig. (2-tailed)	.923	
Morning serum osmolality	Pearson correlation	.092	.353*
	Sig. (2-tailed)	.593	.035
Morning Serum creatinine	Pearson correlation	−.276	−.149
	Sig. (2-tailed)	.089	.386
Morning serum sodium	Pearson correlation	.159	.604**
	Sig. (2-tailed)	.429	.001
Morning serum urea	Pearson correlation	−.035	.181
	Sig. (2-tailed)	.837	.298
Morning serum glucose	Pearson correlation	−.079	−.274
	Sig. (2-tailed)	.631	.106
Morning urinary osmolality	Pearson correlation	−.001	.394*
	Sig. (2-tailed)	.996	.016
Morning urine specific gravity	Pearson correlation	.030	.169
	Sig. (2-tailed)	.860	.348
Morning Ca/Cr ratio in urine	Pearson correlation	.040	.495*
	Sig. (2-tailed)	.858	.022
Morning Alb/Cr ratio in urine	Pearson correlation	−.213	−.423*
	Sig. (2-tailed)	.286	.028

* Correlation is significant at the 0.05 level.

** Correlation is highly significant at the 0.01 level

**Table 4b:** Correlation between night serum AVP, urine AQP-2 and studied parameters in patients.

Variables		Night serumAVP	Night urine AQP-2
Night serum AVP	Pearson correlation	1	.116
	Sig. (2-tailed)		.534
Night urine AQP-2	Pearson correlation	.116	1
	Sig. (2-tailed)	.534	
Night serum osmolality	Pearson correlation	−.312	−.205
	Sig. (2-tailed)	.120	.315
Night serum creatinine	Pearson correlation	−.014	–.354
	Sig. (2-tailed)	.941	.064
Night serum sodium	Pearson correlation	.127	.151
	Sig. (2-tailed)	.497	.394
Night serum urea	Pearson correlation	.050	−.200
	Sig. (2-tailed)	.804	.327
Night serum glucose	Pearson correlation	−.195	−.609**
	Sig. (2-tailed)	.329	.001
Night urinary osmolality	Pearson correlation	.085	.465**
	Sig. (2-tailed)	.653	.006
Night urine specific gravity	Pearson correlation	.163	−.087
	Sig. (2-tailed)	.373	.627
Night Ca/Cr ratio in urine	Pearson correlation	−.035	.215
	Sig. (2-tailed)	.885	.363
Night Alb/Cr ratio in urine	Pearson correlation	.151	−.123
	Sig. (2-tailed)	.503	.551

* Correlation is significant at the 0.05 level.

** Correlation is highly significant at the 0.01 level.
